# Assessing the dynamics and macromolecular interactions of the intrinsically disordered protein YY1

**DOI:** 10.1042/BSR20231295

**Published:** 2023-10-27

**Authors:** Heather Donald, Ashleigh Blane, Sindisiwe Buthelezi, Previn Naicker, Stoyan Stoychev, Jacob Majakwara, Sylvia Fanucchi

**Affiliations:** 1 Protein Structure-Function Unit, School of molecular and Cell Biology, University of the Witwatersrand, Jan Smuts Ave, Braamfontein, 2050 Johannesburg, Gauteng, South Africa; 2 CSIR Biosciences, CSIR, Meiring Naude Road, Brummeria, 0001 Pretoria, Gauteng, South Africa; 3 School of Statistics and Actuarial Science, University of the Witwatersrand, Jan Smuts Ave, Braamfontein, 2050 Johannesburg, Gauteng, South Africa

**Keywords:** DNA binding, forkhead proteins, hydrogen-deuterium exchange mass spectrometry, intrinsically disordered proteins, protein-protein interactions, YY1

## Abstract

YY1 is a ubiquitously expressed, intrinsically disordered transcription factor involved in neural development. The oligomeric state of YY1 varies depending on the environment. These structural changes may alter its DNA binding ability and hence its transcriptional activity. Just as YY1’s oligomeric state can impact its role in transcription, so does its interaction with other proteins such as FOXP2. The aim of this work is to study the structure and dynamics of YY1 so as to determine the influence of oligomerisation and associations with FOXP2 on its DNA binding mechanism. The results confirm that YY1 is primarily a disordered protein, but it does consist of certain specific structured regions. We observed that YY1 quaternary structure is a heterogenous mixture of oligomers, the overall size of which is dependent on ionic strength. Both YY1 oligomerisation and its dynamic behaviour are further subject to changes upon DNA binding, whereby increases in DNA concentration result in a decrease in the size of YY1 oligomers. YY1 and the FOXP2 forkhead domain were found to interact with each other both in isolation and in the presence of YY1-specific DNA. The heterogeneous, dynamic multimerisation of YY1 identified in this work is, therefore likely to be important for its ability to make heterologous associations with other proteins such as FOXP2. The interactions that YY1 makes with itself, FOXP2 and DNA form part of an intricate mechanism of transcriptional regulation by YY1, which is vital for appropriate neural development.

## Introduction

Neurological disorders such as autism spectrum disorder result from the misregulation of complex transcriptional pathways. Tight transcriptional control, including the control of processes such as neural development, is a necessity for any developmental or homeostatic process. It requires the involvement of a complex and highly controlled network consisting of transcription factor–nucleic acid interactions. There are many mechanisms of control available to transcription factors, and a single transcription factor may be capable of several of these [1–4]. This flexibility in the mechanism of action of transcription factors can be attributed, in part, to the high proportion of intrinsically disordered structure either in the entire protein or in large regions of the transcription factor [5–7].

Despite the abundance of intrinsically disordered proteins within the proteome, their significance in relation to function has only been explored relatively recently [5,7–9]. Intrinsically disordered proteins play a vital role in gene expression through their highly dynamic nature and their unique abilities to form multiple protein–protein and protein–DNA interactions, allowing many to act as hubs for transcription factor associations [5,10,11]. One such case is the formation of proteinaceous membrane-less organelles. These structures form when high concentrations of protein and nucleic acids undergo liquid–liquid phase transitions [6,9,12,13]. Intrinsically disordered proteins contribute to the formation of proteinaceous membrane-less organelles through their ability to bind to multiple proteins and other macromolecules, thereby recruiting them into the organelle [6,14]. Membrane-less organelles form various functional structures in the cell, including nucleoli, nuclear speckles (involved in RNA processing) and polycomb bodies (involved in transcriptional repression) [6,15–17]. Certain transcription factors have been identified to play important roles in the formation of proteinaceous membrane-less organelles [6,14,18,19]. One such transcription factor is Ying Yang 1 (YY1), which has been identified in the recruitment of polycomb proteins [14,18], and also in nuclear speckles [20]. Indeed, recently YY1 has been demonstrated to be crucial in driving phase separation required for transcriptional activation [19,21].

YY1 is a ubiquitously expressed transcription factor which has been identified as an intrinsically disordered protein [22]. Consequently, most of its sequence is disordered but some structured regions are present [22]. The sequence consists of two broad regions: the disordered N-terminal region and the more structured C-terminal region that contains four C2H2 zinc finger motifs which make up the YY1 DNA binding domain [23]. This domain is also able to bind to RNA and other proteins [24]. The mostly disordered N terminal region contains two acid rich regions, a polyhistidine region, a GA- and a GK-rich region, as well as the spacer region [25]. The spacer region exists between the N-terminal and C-terminal regions and contains two sections which are conserved across YY1 homologs, but do not share similarity with any other protein [26]. One of these regions forms a beta turn that was previously referred to as the recruitment of polycomb (REPO) domain [18]. The structure of the N-terminal region, while confirmed to be mostly disordered and also important in phase separation [19,22], has not been examined in detail. Sequence-based predictions suggest that there is propensity for the formation of pockets of structure in the form of beta strands within this region [22]; however, this has not been confirmed.

YY1 is involved in a wide range of processes, including neural development [27–32]. More specifically, YY1 has been associated with DNA structuring [33,34]; transcriptional initiation [4], activation and repression [21,25,33,35,36], DNA repair [37], and the formation of the polycomb group [14,18] and nuclear speckles [20]. Haploinsufficiency of YY1 results in Gabriel-de Vreis syndrome, the symptoms of which are broad and primarily include developmental delay and intellectual disabilities such as autism spectrum disorder and language impairment [27,38]. Additionally, misregulation of YY1 has been associated with the neurodegenerative conditions Alzeheimer’s [39] and Parkinson’s disease [40], as well as the development of various cancers [41–44].

The involvement of YY1 in this wide range of functional processes is likely linked to its intrinsically disordered nature, where its behaviour and the homologous and heterologous interactions that it forms are highly dependent on the changing conditions within the cell [6,7]. YY1 has been found to form oligomers which are capable of binding to DNA [33,37,45]. However, the literature concerning the formation of YY1 oligomers is disparate, where some studies have observed a mixture of YY1 oligomeric species in solution (potential dimers, tetramers and higher order oligomers) [37,45], while others report only a single monomer [22,46]. *In vivo*, YY1 self-association at DNA looping regions has been observed [33]. Under specific conditions, it has been demonstrated to form phase-separated condensates [19,21]. The conditions influencing YY1 multimerisation, the role of various specific oligomeric species in YY1 function and the effect of YY1 oligomerisation on DNA binding have not been explored. We, therefore, investigated YY1 self-association in this work by determining whether ionic strength influences YY1 oligomerisation and at what protein concentrations this influence is most pronounced. Knowing the conditions that influence the oligomerisation of this protein will allow us to better understand its transcriptional mechanism.

In addition to environment-induced modifications to YY1 structure, the mechanism by which it regulates transcription is also heavily influenced by heterologous protein–protein interactions [14,24,25,37,45,47]. Due to its flexible and intrinsically disordered nature, YY1 associates readily with other proteins. A number of YY1 binding partners have been identified, and they appear to influence YY1 transcription in various ways including by increasing its affinity for certain DNA sequences [45]. Such binding partners include RNA polymerase [4], histone modifying proteins such as HDAC2 [25,48], DNA repair proteins of the INO80 complex [37] and proteins involved in the cell cycle such as p53 and Hdm2 [49].

FOXP2 (forkhead box P2) is a transcription factor which operates in similar pathways to YY1, and is involved in neural and, more specifically, language development [50–53]. FOXP2, like YY1, contains pockets of structure and stretches of disordered sequence. It contains a long N-terminal polyQ tract, followed by zinc finger and leucine zipper domains. The DNA binding region is located at the C-terminal end of the sequence. It is a winged helix motif referred to as the forkhead domain (FHD) [54]. YY1 and FOXP2 have been identified as potential interaction partners due to their co-expression in the cerebral cortex [55]. YY1 and FOXP2 were found to form a physical interaction through cell lysate affinity assays and in vivo BRET assays [55]. From this study, it was established that YY1 potentially interacts with more than one region of FOXP2. However, detailed and important information about the direct association between these two proteins, including where and how they interact, how the interaction is regulated, as well as how the interaction may influence their respective functions, is unknown. A better understanding of the structure and function of this protein–protein interaction will help to further clarify the complex neuromolecular processes in the brain which contribute to language and cognition and will also help us better understand how YY1 controls processes such as neural development.

The aim of the present study is, therefore, to explore the network of interactions that YY1 forms with (a) itself, (b) DNA and (c) the FOXP2 FHD so as to obtain definitive answers on where these interactions occur and how they influence each other.

## Results

We studied the structure and dynamics of YY1 interactions by dividing them into three groups. YY1 self-association, YY1 DNA binding and YY1–FOXP2 protein–protein interactions.

### YY1 self-association

In order to observe how YY1 oligomerisation might be influenced by different conditions, we utilised two methods: SE-HPLC and fluorescence anisotropy. SE-HPLC was used to provide an overall profile of YY1 quaternary structures present under differing conditions while the anisotropy was used as a supplement, to observe the overall size shift of the population more easily. The conditions which were investigated included changes in both protein concentration and ionic strength. This was done in order to see if YY1 self-association is governed by electrostatic contacts, and to determine at which protein concentrations YY1 formed multimers.

**Figure 1:**
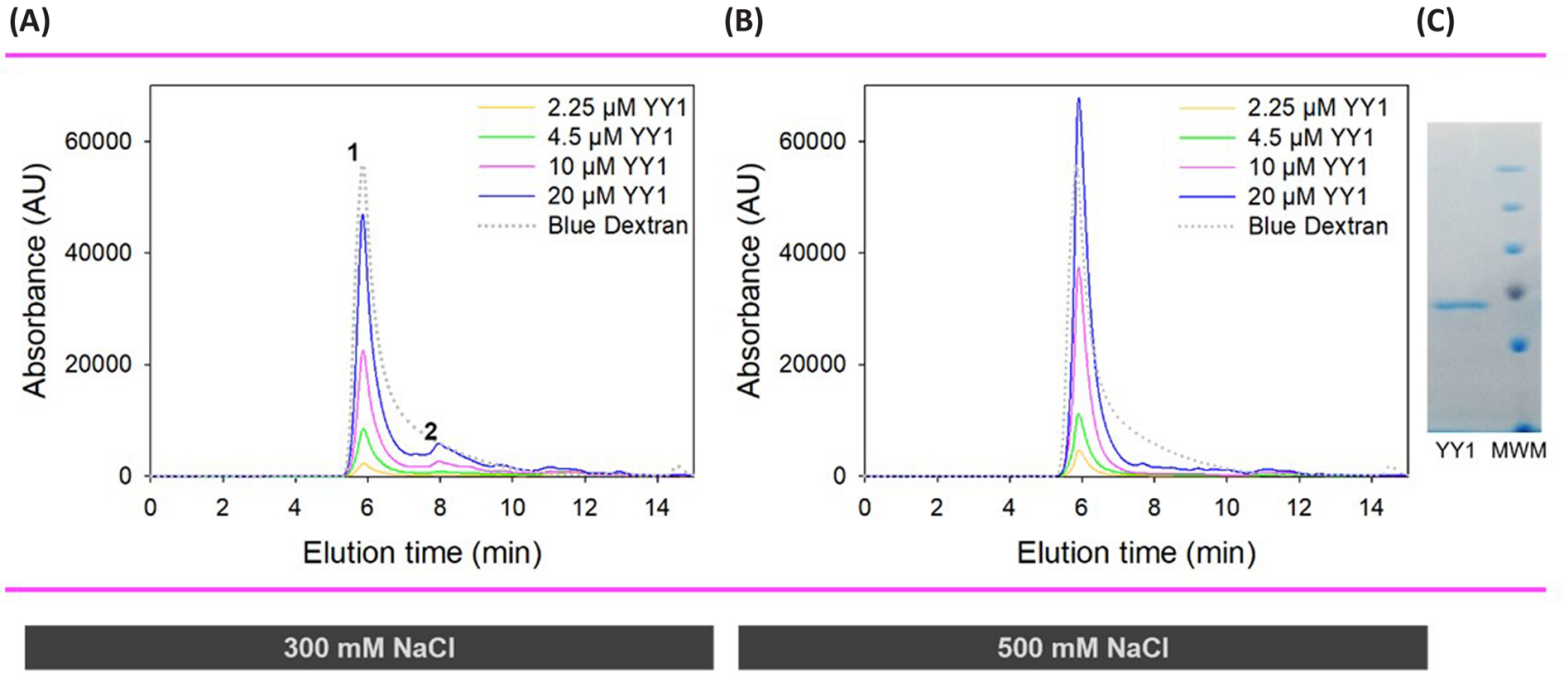
SE-HPLC profiles for YY1 under low (300 mM) and high (500 mM) salt conditions (**A**) SE-HPLC elution profile showing increasing concentrations (2.25–20 μM) of YY1 at 300 mM NaCl (**B**) SE-HPLC elution profile showing increasing concentrations (2.25–20 μM) of YY1 at 500 mM NaCl. On average, there is a greater proportion of large multimers at the higher salt concentration. Blue dextran indicates the void volume. There is at least one pronounced second peak apparent in the samples containing lower salt concentrations, indicating that YY1 can exist in a mixture of oligomeric forms of various sizes. Figures were plotted in SigmaPlot 12.0. (**C**) SDS PAGE gel of the pure YY1 fraction used in the SE-HPLC samples.

It is clear from the size exclusion chromatography elution profile (Figure 1), that at all protein concentrations investigated (2.5–20 µM) and at both 300 and 500 mM NaCl, YY1 exists mostly as large oligomers – likely to be a heterogenous mixture of various sizes – that elute in the void volume (larger than 600 kDa) (Peak 1). However, a low proportion of smaller species is also detectable only at the lower salt concentration (300 mM NaCl). This shift to a greater proportion of smaller species being populated at lower ionic strength indicates that lower salt reduces YY1 self-association (Figure 1).

Given the intrinsically disordered (non-globular) nature of YY1, it is difficult to assess the size of any of the peaks in Figure 1 with confidence as the larger hydrodynamic volume will overestimate the size compared with the globular standards. However, from a qualitative perspective, it is clear that the smaller peak (peak 2) is not well resolved, which may imply that it results from transient YY1–YY1 interactions. Peaks 1 and 2 have also been observed in previous SE-HPLC studies at 200 mM NaCl in varying proportions, indicating consistency of our data with that reported by others [37,45]. An electron microscopy assessment of the different species suggested the presence of both dimer and trimer species, as well as large irregular aggregate-like bodies [45].

**Figure 2:**
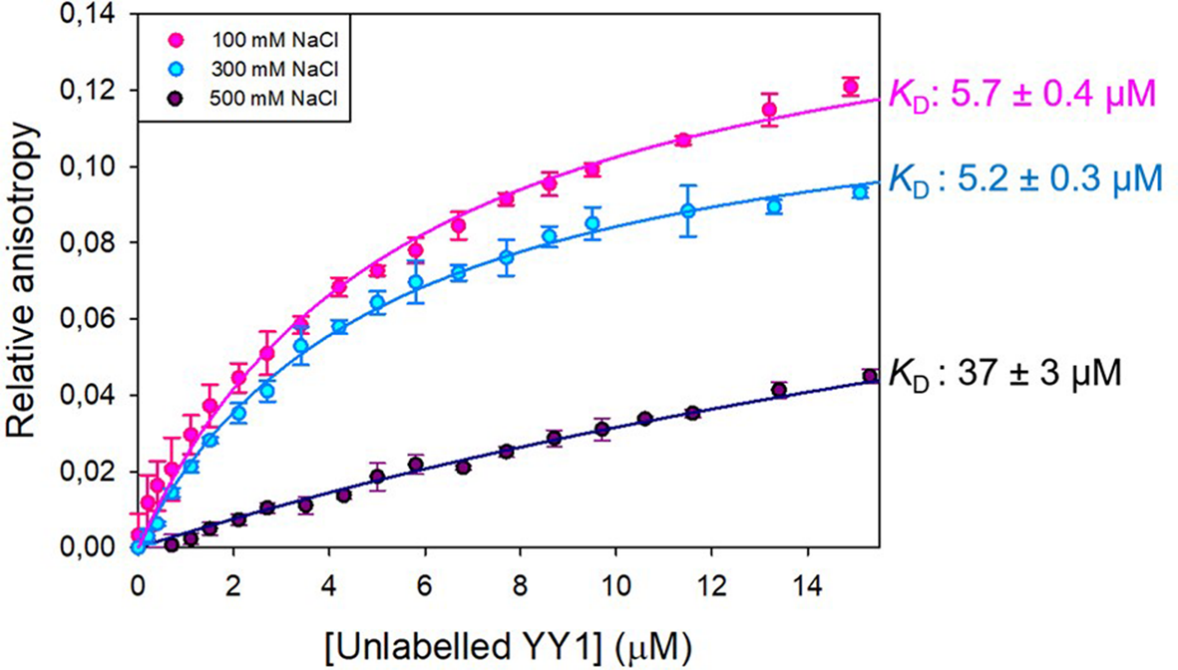
Anisotropy binding curves showing YY1–YY1 interaction at different salt concentrations Unlabelled YY1 was titrated against 0.2 μM YY1 labelled with NTA ATTO 550. The pink, blue and black curves show YY1 self-association in a buffer containing 100, 300 or 500 mM NaCl respectively. (*R*^2^ = 0.98 for 100 mM and 500 mM NaCl and 0.99 for 300 mM NaCl). On the right are the predicted apparent *K*_D_ values for each curve. The curve was made and fitted (to a ligand binding single site model) using SigmaPlot 12.0.

Given the size exclusion limitations in observing species in the void volume, we did a supplementary anisotropy assay to better probe the average YY1 oligomeric size shift upon the same varying conditions: concentration and ionic strength (Figure 2). This was achieved by titrating unlabelled YY1 into YY1 labelled with NTA ATTO-550 at the N-terminus. A single site binding model was fitted to the data (Figure 2). This model was selected based on the assumption that maximum binding indicates the occupation of all binding sites. Because of the heterogeneity of YY1 oligomers in solution, the dissociation constant cannot be accurately interpreted as a measure of affinity. The *K*_D_ obtained from this fit does not represent the concentration of YY1 at which half the species are monomeric and half the species are dimeric. However, we can instead define the ‘apparent’ *K*_D_ to be the concentration of YY1 at which the average oligomeric profile of YY1 shifts from consisting predominantly of lower order species to consisting predominantly of higher order species. This allows us to conclude definitively that YY1 oligomerisation is affected by salt, with a significant difference being observed between 300 and 500 mM NaCl (Dunn test: *P*=0.00).

Together, the anisotropy and SE-HPLC results indicate that YY1 self-associates to form large oligomers with an apparent *K*_D_ in the low micromolar range, and that this association is influenced by ionic strength. Higher ionic strength results in a shift in the equilibrium towards larger YY1 species. The increase in apparent *K*_D_ seen at 500 mM NaCl from the anisotropy study, does not necessarily suggest a decrease in affinity of YY1 for itself at high salt concentrations. When taken together with the SE-HPLC results, we can conclude that the shift in apparent *K*_D_ at high salt is due to the smaller difference in size between the first titration and final saturation. Because we know that high salt conditions cause a shift towards larger YY1 species (Figure 1), it is likely that the anisotropy curves measured at different salt concentrations (Figure 2) start at different points along the isotherm in terms of average oligomer size. In other words, although all three curves started with 0.2 µM YY1, at the higher salt concentration, on average, there will be larger oligomers already present under these conditions than at 300 mM NaCl. This means that the starting point for the 500 mM YY1 titration curve is already closer to saturation than the corresponding points in the other two curves. At lower concentrations of NaCl, on the other hand, increases in YY1 concentration have a greater effect on the oligomeric profile of YY1, because smaller species are present initially, but the final saturation size is the same.

### YY1 DNA binding and its effect on YY1 self-association

**Figure 3:**
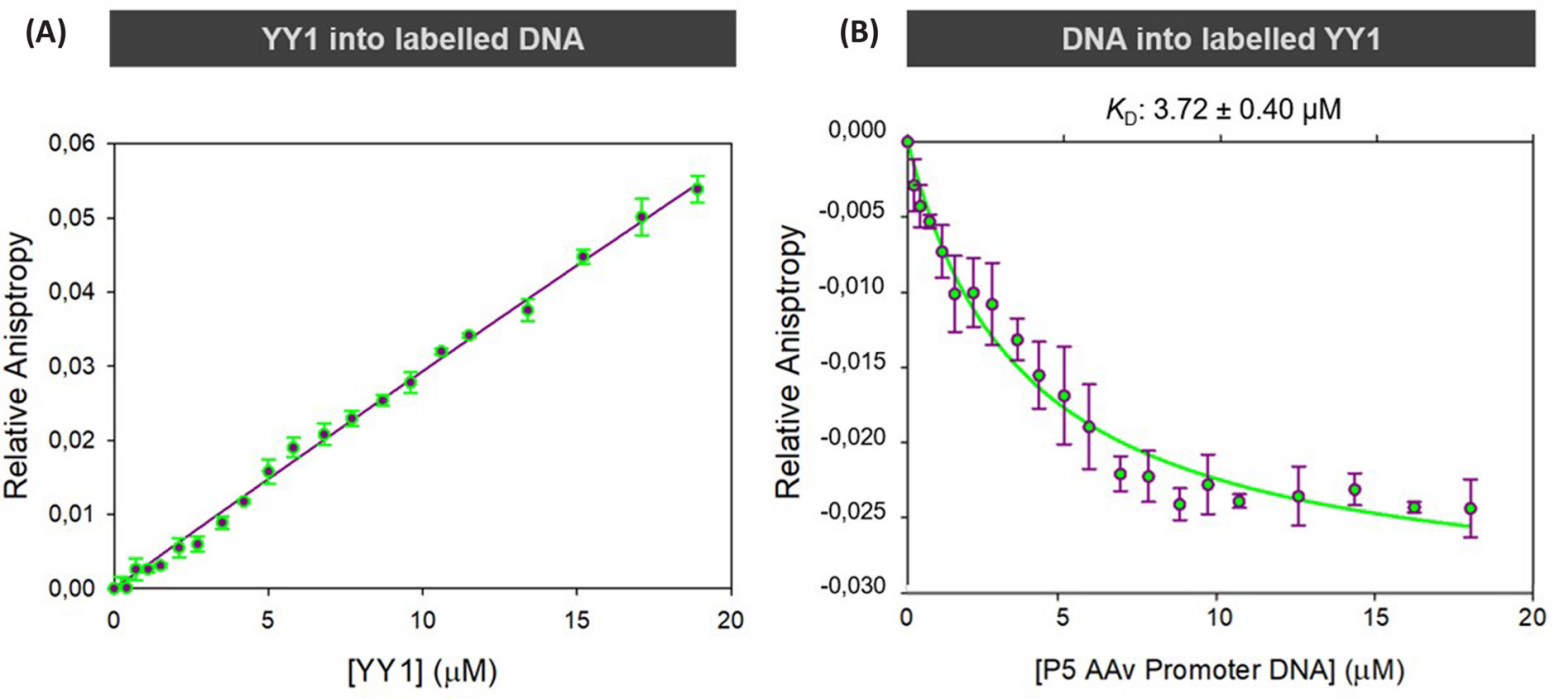
YY1 binding to DNA as seen with fluorescence anisotropy (**A**) YY1–DNA binding curve showing YY1 titrated against 1 µM ROX-labelled AAV P5 DNA (*R*^2^ = 0.99). (**B**) YY1–DNA binding curve showing AAV P5 DNA titrated against 0.2 µM YY1 labelled with NTA ATTO 550 (*R*^2^ = 0.93). While YY1 is able to self-associate to form larger oligomers when bound to DNA at low concentrations, saturating concentrations of DNA result in decreased size of the YY1 oligomers. Both anisotropy studies were performed in the presence of 100 mM NaCl. Graphs were generated using SigmaPlot 12.0.

Understanding the dynamic between YY1 multimerisation and its binding to DNA could help to explain how YY1 oligomers may be involved in transcriptional control. Fluorescence anisotropy was used to investigate the binding of YY1 to DNA. Figure 3 shows the YY1–DNA binding curves obtained using fluorescence anisotropy at 100 mM NaCl [56].

The titration of YY1 against ROX-labelled DNA (Figure 3A) does not reach saturation. Unfortunately, higher concentrations than those used could not be achieved without compromising protein stability. Because YY1 can self-associate, it is likely that the observed lack of saturation is due to YY1 oligomers assembling on the DNA. Since the DNA is labelled, we are only detecting the increase in size of the DNA-bound complex. However, this increase in size is not simply from protein binding to DNA but also from protein binding to protein that is already bound to DNA.

When DNA is titrated against 0.2 µM YY1 labelled with NTA ATTO-550 (Figure 3B), there is an overall decrease in complex size with increasing DNA concentration as indicated by the decreasing relative anisotropy value. This size decrease suggests that saturating amounts of DNA can disrupt some of the large YY1 oligomeric complexes, forming YY1–DNA complexes that are smaller than the YY1 oligomer that the DNA was originally titrated against. As a complete isotherm was achieved in Figure 3B, an apparent *K*_D_ for DNA binding of 3.7 ± 0.4 µM could be calculated. Together, these results show how the oligomeric state of YY1 is influenced by the presence of DNA, where at low DNA concentrations YY1 is able to form large oligomers bound to the DNA, but excess DNA will cause a decrease in the size of the YY1 oligomer.

### YY1 dynamics in the presence and absence of DNA

**Figure 4:**
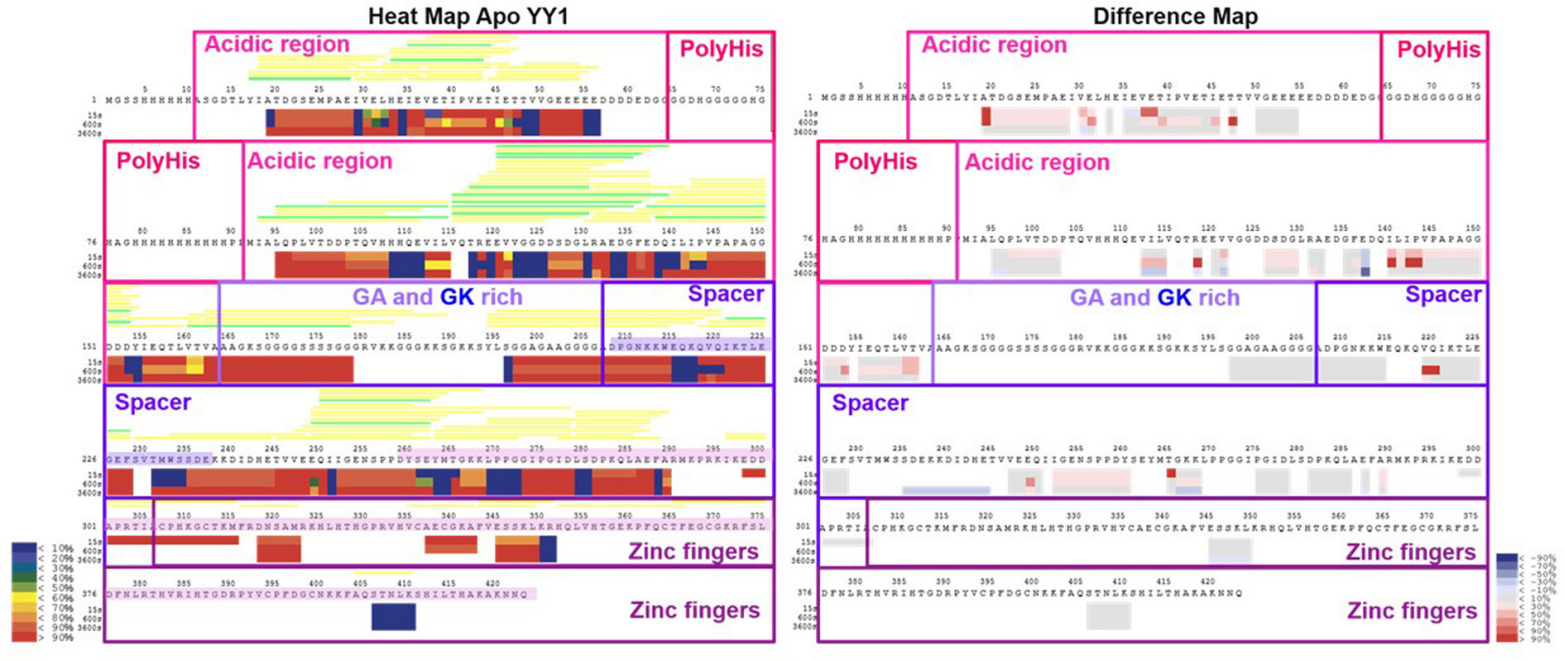
Heat map and difference map of YY1 backbone dynamics as generated by HDXMS **Left**: Heat map showing the % of deuteration of the backbone amide for each residue as represented by the colour scale, ranging from red (more than 90% deuteration) to blue (less than 10% deuteration). The less deuterated a residue’s backbone is, the more likely it is to be buried within the structure, whereas the more highly deuterated residue backbones are more exposed to the solvent. Since most of YY1 backbone is highly deuterated, it implies that it has a flexible and open structure. The green and yellow bars above the heat map represent the high and medium confidence peptides used to create the heat map. Purple highlighted amino acid sequence shows the location of the REPO domain within the spacer region, while pink highlighted sequence indicates conserved sections of YY1. **Right**: The difference map shows the difference in deuterium uptake between the DNA bound and apo forms. The difference map is coloured on a scale from red to blue, where red shows a greater uptake of deuterium and blue is a decrease in deuterium uptake upon DNA binding. The greatest differences are within the acidic region. The heat map shows that YY1 is predominantly disordered, with a few specific residues which are likely to form pockets of structure. These are especially prominent in the acidic region and conserved areas of the spacer. Heat maps were generated using HD Examiner (Sierra Analytics, U.S.A.).

**Figure 5:**
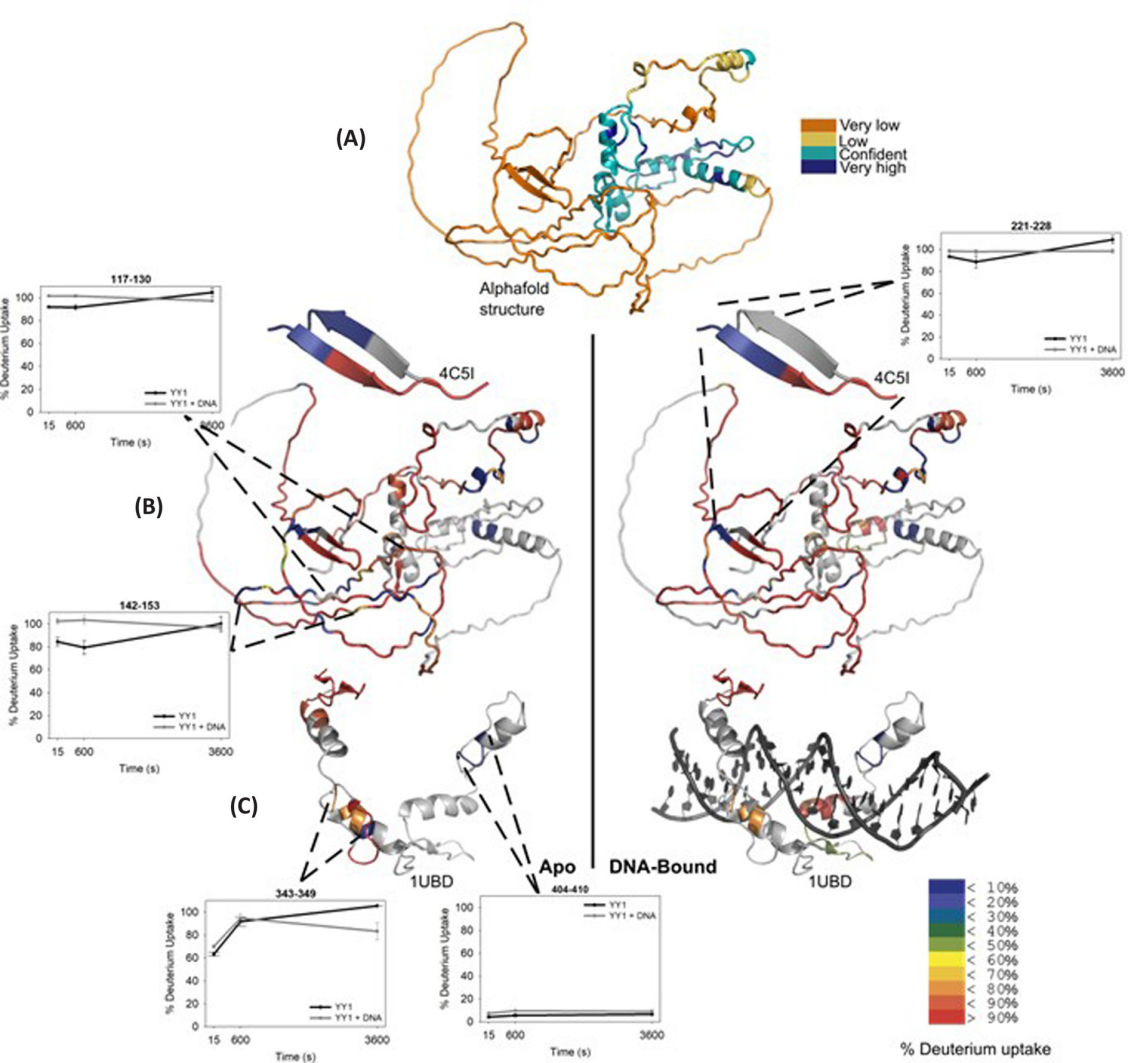
Structural representation of YY1 and its dynamics (**A**) The structure of full length YY1 as predicted by Alphafold2 [59,60], coloured by the confidence in the model. Lower confidence (low-very low) is typically associated with disorder, and is therefore not an accurate reflection of the structure depicted. (**B**) The structure coloured by deuterium uptake percentages. Loops that, according to the uptake plots, appear to have more structure than Alphafold2 suggests, might represent protein–protein interface. The beta turn in the Alphaold2 figure corresponds the REPO domain which has already been crystallised (PDB: 4C5I). (**C**) The crystal structure of the YY1 DNA binding domain is shown (PDB: 1UBD), coloured by the percentage of deuterium uptake (shown on the bottom right). Structures on the left depict the apo YY1 at 15 s deuteration, and on the right is the respective DNA-bound form. Typical deuterium uptake plots for regions in the N-terminal, DNA binding domain and REPO motif are shown.

HDXMS was employed to assess YY1 backbone dynamics when in the apo form or when bound to ∼3× molar excess DNA. This was done to better understand the mechanisms behind the interplay between DNA binding and oligomerisation of YY1. Briefly, the peptides measured for deuterium uptake were used to put together a heatmap showing deuterium uptake across the YY1 sequence, where regions of peptide overlap result in higher resolution for that section of sequence. Deuterium uptake levels, and by extension, flexibility of the protein, are expressed by heatmap colours (Figures 4 and 5).

**Figure 6:**
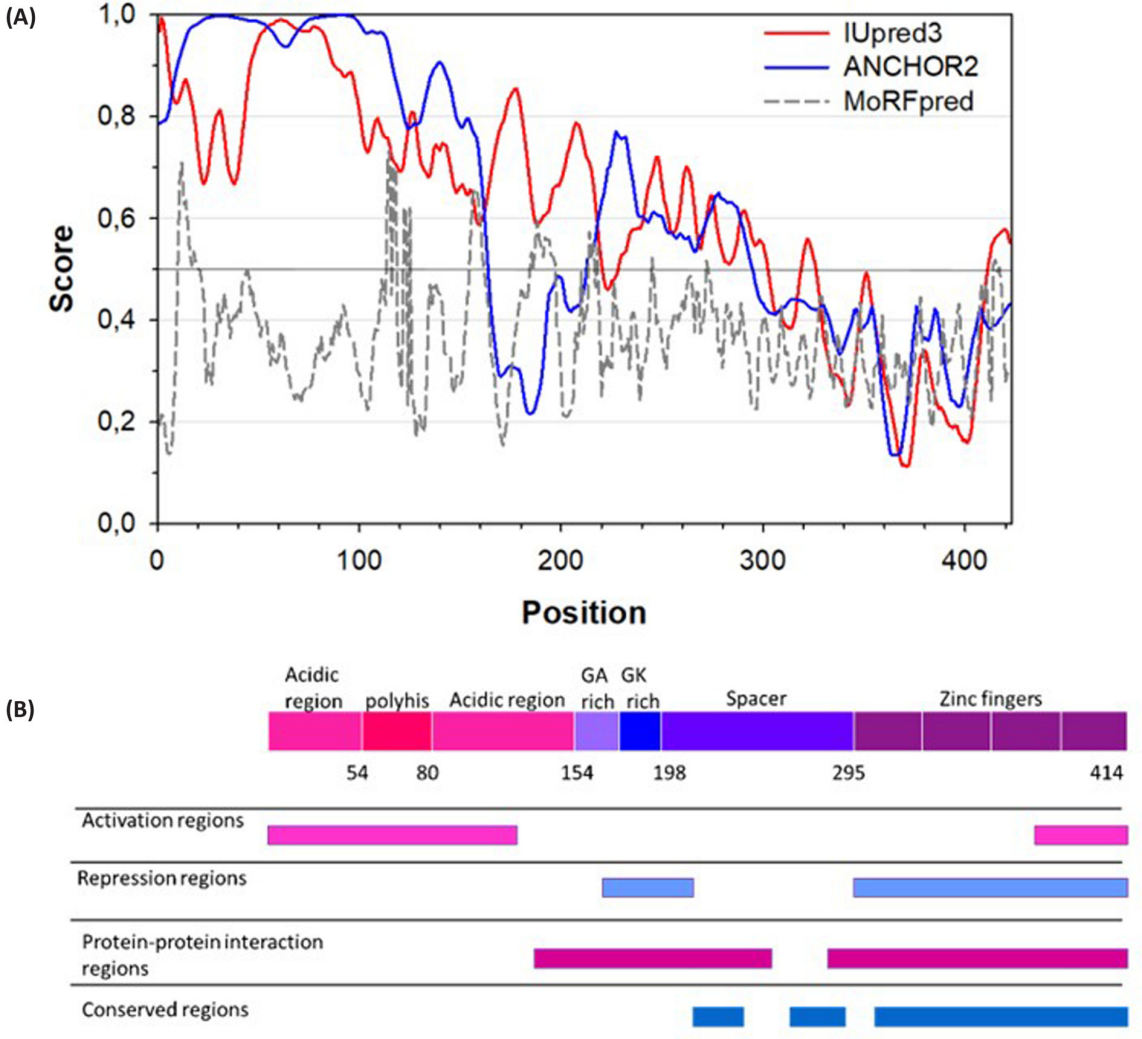
Disorder, MoRF prediction and sequence summary for YY1 (**A**) Predicted ordered/disordered regions across the YY1 sequence as analysed by IUpred3 (red). Blue indicates the ANCHOR2 prediction of regions which may form structure upon binding to other proteins. Similarly, grey indicates the MoRFpred analysis of YY1, where a higher score means a more likely MoRF. Scores above 0.5 indicate disorder for the IUpred analysis, while for the ANCHOR and MoRF analyses a higher score indicates more likely formation of structure upon binding. (**B**) Structural diagram of sections of YY1’s sequence and the characteristics with which those regions are strongly associated. The N-terminal portion of the protein is more likely to become structured upon the formation of protein-protein interactions.

In the apo form, YY1 was observed to be overall highly exposed to solution across all time points, reflecting its intrinsically disordered nature (Figures 4 and 5). However, as predicted, the protein was not entirely disordered, and pockets of non-exposed sequence were observed. These were especially prominent in the acidic regions (residues 10-63 and 92-163, towards the N-terminal end), and may reflect sites which have induced structure due to self-association. Indeed, the ANCHOR2 [57] and MORFpred [58] predictions of regions which may gain induced structure in a binding event (Figure 6) correspond to these patches of low uptake.

Sections of the spacer region (residues 207-304) – mostly conserved sections – contained stretches of residues with folded structure, as indicated by their lack of deuterium uptake (residues 215-220, 231-234, 261-272 and 279-280). Unfortunately, very little peptide coverage of the zinc finger region was obtained, and so few conclusions could be made about YY1’s dynamic behaviour in the DNA-binding domain (DBD) (residues 304-end).

Despite the lack of data pertaining to the DBD of YY1, other regions of the protein were identified as experiencing changes in dynamics upon DNA binding. The most distinct changes were observed in the acidic regions, where the most frequently observed trend was an increase in the exposure of the backbone to solution (deuterium uptake change of 50% was taken as large, and no changes smaller than 30% were considered) (Figures 4 and 5). As such, it appears that the acidic regions experience an increase in disorder upon DNA binding. Considering DNA binding decreases the propensity of the protein to form oligomers (Figure 3), this increase in flexibility of the acidic region may also reflect a disruption of a homologous protein-protein interaction. This is supported by predictions of structural formation upon protein–protein interactions with the ANCHOR and MoRFpred analyses (Figure 6). Changes in dynamics between apo and DNA-bound protein were also observed in sections of the spacer region, although to a lesser extent.

### YY1–FOXP2 FHD interaction

**Figure 7:**
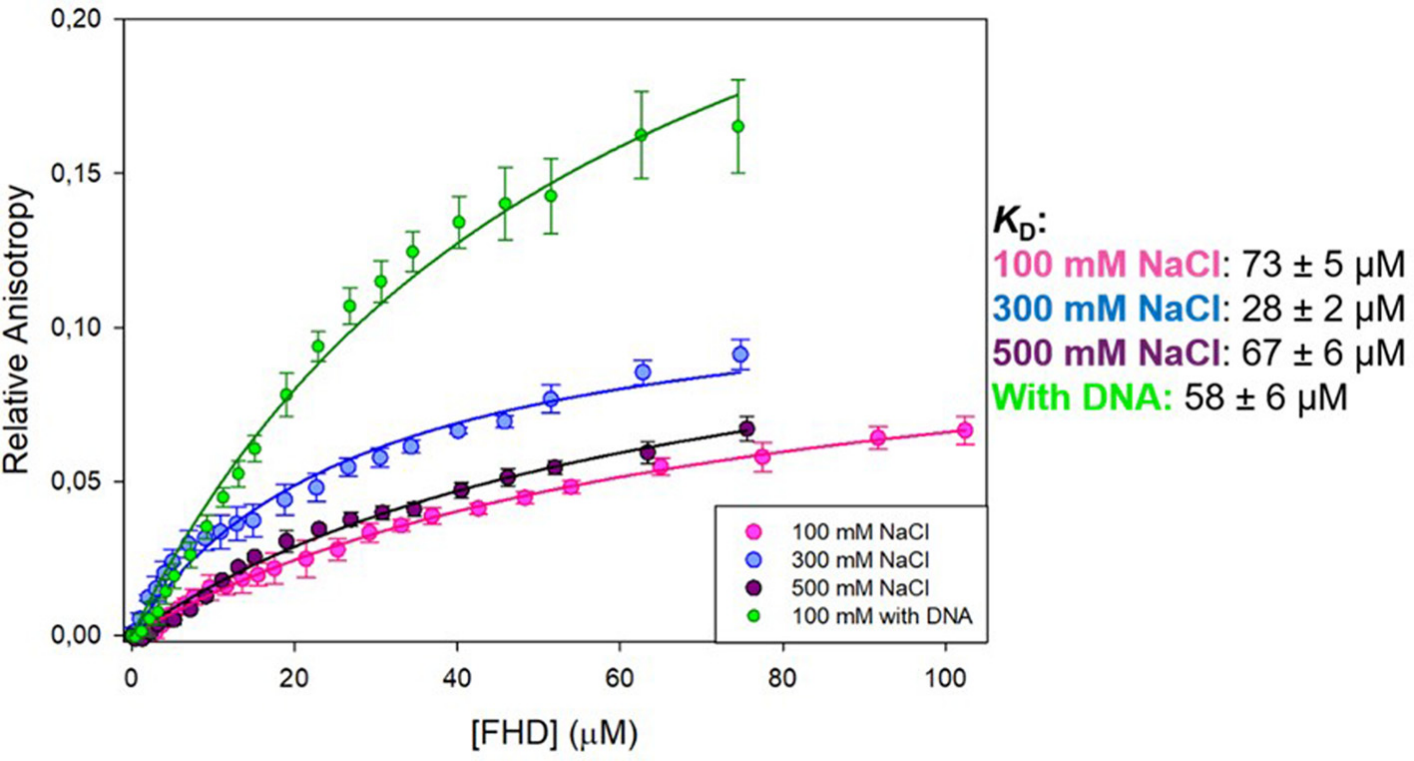
FOXP2 FHD-YY1 binding curves using fluorescence anisotropy Fluorescence anisotropy assay of 0.2 μM YY1 labelled with NTA ATTO550, with increasing concentrations of FOXP2 FHD titrated against it. Each curve shows a different salt condition, where pink is YY1 in the presence of 100 mM NaCl (*R*^2^ = 0.98), blue is YY1 in 300 mM NaCl (*R*^2^ = 0.97), and purple is YY1 in 500 mM NaCl (*R*^2^ = 0.98). No significant difference in the binding curves was found (one-way ANOVA; *P*=0.05318). The green curve represents YY1 bound to 10 μM YY1-specific DNA in the presence of 100 mM NaCl (*R*^2^ = 0.98). YY1 does bind to the FOXP2 FHD *in vitro*. This binding is independent of both the salt concentration and the presence of DNA. Graphs were plotted using SigmaPlot 12.0.

YY1 and FOXP2 are involved in similar pathways of neural development, and both are associated with speech disorders and autism spectrum disorder [27,28,50,51]. These two proteins have been found in proximity *in vivo* [55], but whether a direct association occurs, and how this may affect their respective functions, has not previously been assessed. To determine if YY1 and FOXP2 FHD bind to each other in isolation and if this interaction is electrostatic in nature, fluorescence anisotropy was used at three concentrations of NaCl: 100, 300 and 500 mM (Figure 7). Specifically, unlabelled FOXP2 FHD was titrated into YY1 labelled with NTA ATTO 550. FOXP2 FHD was chosen to be titrated against labelled YY1 due to the relatively higher stability of FOXP2 FHD at high concentrations compared with YY1, as well as due to the capacity of YY1 to be labelled efficiently in comparison with FOXP2 FHD. A single ligand binding model was fitted to the data so as to calculate the apparent *K*_D_. The calculated apparent affinities of YY1 for FOXP2 FHD were approximately 73, 28 and 67 μM when in the presence of 100, 300 and 500 mM NaCl, respectively. This indicates that there is an association between YY1 and FOXP2 FHD in isolation. This interaction is relatively weak and does not appear to be significantly affected by salt in the range of 100–500 mM (ANOVA; *P*=0.05318), indicating that the association between the two proteins is not governed by electrostatics and the oligomeric state of YY1 does not influence its affinity for FOXP2 FHD.

An additional anisotropy titration of FOXP2 FHD against labelled YY1 was performed by including 10 μM background AAV P5 promoter DNA (YY1 specific) throughout the titration. This DNA concentration was enough to saturate YY1. The YY1–DNA interaction was given time to reach equilibrium before FOXP2 FHD was added. This was done so as to investigate whether the addition of DNA altered the interaction between YY1 and FOXP2 FHD (Figure 7). This is an important consideration since all three species would be likely to associate with each other at any given time during transcription. The resulting *K*_D_ was determined as 58 ± 6 μM, which was within the same range as in the absence of DNA. However, a greater change in the overall complex size was observed in the presence of DNA, which may have been due to a decrease in average YY1 complex size upon DNA binding.

**Figure 8:**
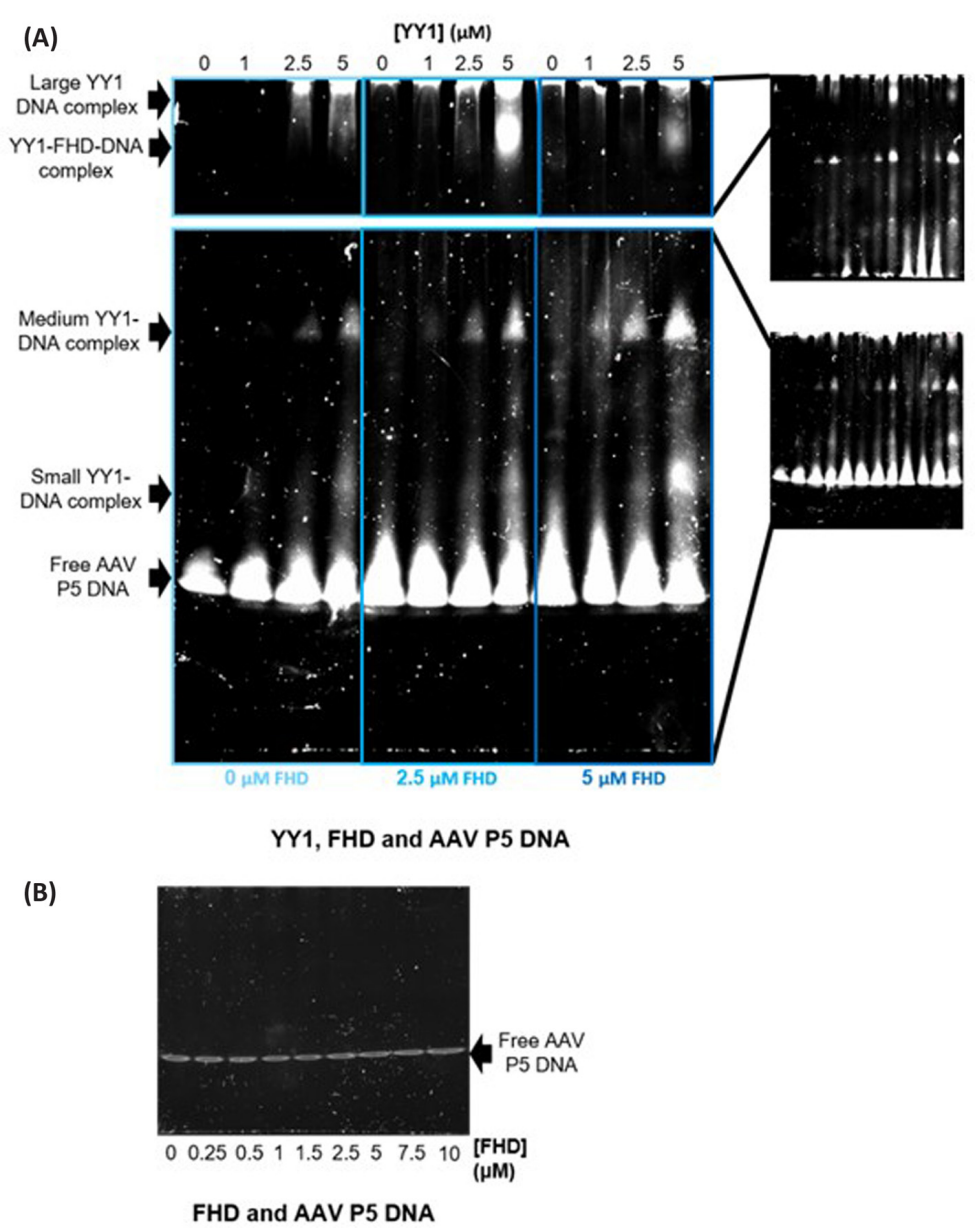
Supershift assay to determine the effects of the FOXP2 FHD on the YY1-AAV P5 DNA interaction (**A**) This figure is made up of two gels which were resolved under exactly the same conditions using the same samples, but run for different times. The top section shows large complexes which required a longer time for resolution, resulting in the free DNA bands no longer having full visibility. The lower section is the corresponding gel with shorter running times, allowing better visibility of smaller species including the free-DNA bands. From the left to the right, each box contains a repeat of identical increasing YY1 concentrations across the lanes. As the boxes move from left to right, the concentration of the second binding partner, FOXP2 FHD, is increased. Within each box, the FOXP2 FHD concentration is kept constant. The concentration of AAV P5 DNA was kept constant throughout. From the gel it can be seen that YY1 forms more than one oligomeric species when bound to DNA and the formation of these oligomeric species is influenced by the presence of FOXP2 FHD. (**B**) Control gel to determine that FOXP2 FHD does not specifically bind to AAV P5 DNA.

To further investigate if and how the YY1–FOXP2 FHD interaction might affect each of the proteins’ respective DNA binding capabilities, two supershift EMSA assays were performed. The first one (Figure 8), investigated the effect of FOXP2 FHD on the interaction between YY1 and its specific DNA sequence (AAV P5 DNA). This interaction was assessed by adding increasing concentrations of FOXP2 FHD to a small range of YY1–DNA complexes. In the absence of FOXP2 FHD, YY1 forms up to three different sized YY1–DNA complexes, with the smaller sized complexes increasing in proportion as DNA concentration increases, consistent with the anisotropy results (Figure 3). Upon the addition of FOXP2 FHD, there was a shift in both position and intensity of the large YY1–DNA species at the top of the gel. This band became more intense and shifted slightly further down with the further addition of FOXP2 FHD. This suggests that YY1 may bind to the FOXP2 FHD and DNA simultaneously, which is supported by the similar apparent *K*_D_ found in the YY1–FHD–DNA fluorescence anisotropy assay (Figure 7).

**Figure 9:**
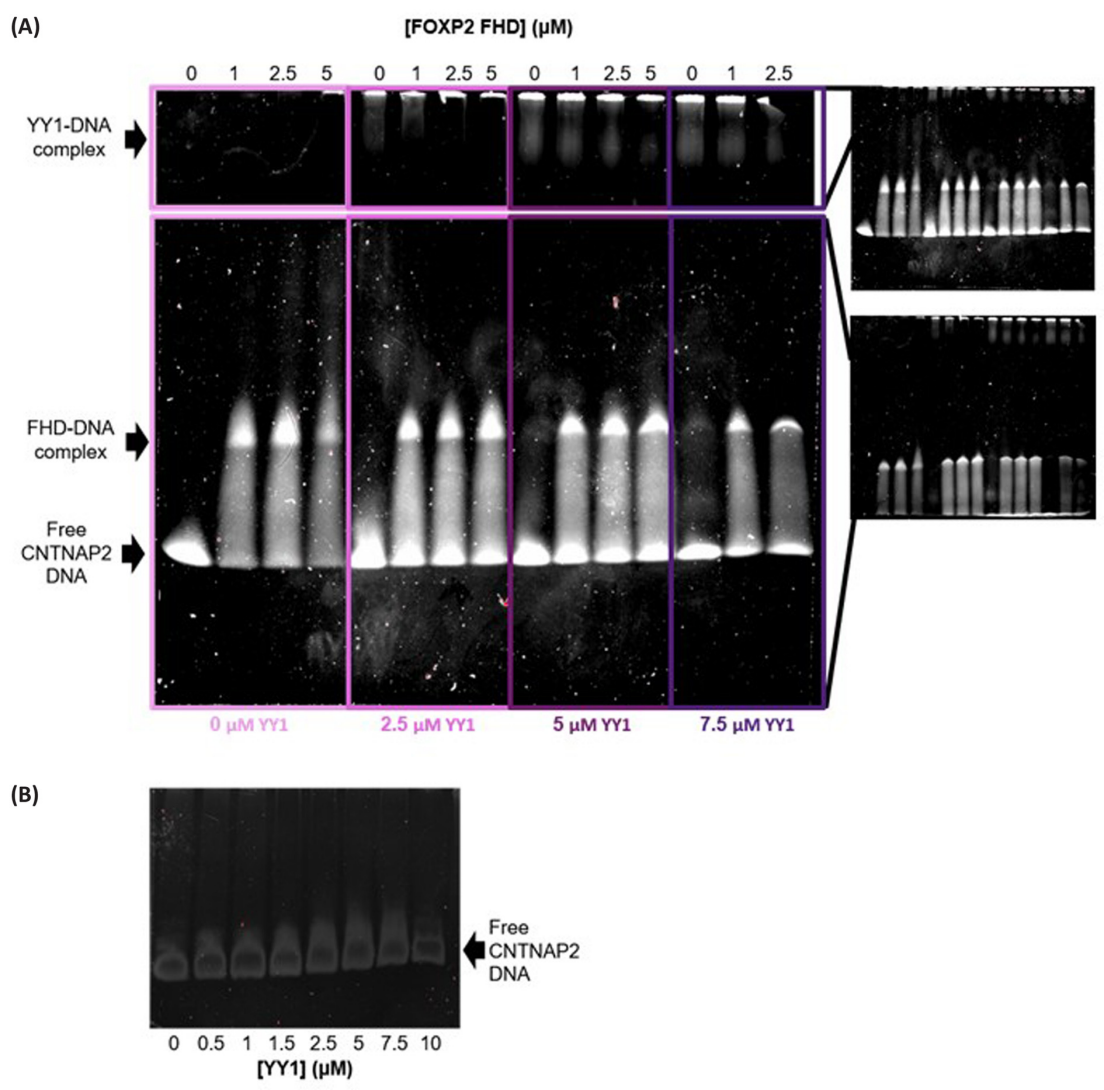
Supershift assay showing the influence of YY1 on the FOXP2 FHD-CNTNAP2 DNA interaction (**A**) This figure is made up of two gels which were resolved under exactly the same conditions using the same samples. The top section shows large complexes which required a longer time for resolution, resulting in the free DNA bands no longer having full visibility. The lower section is the corresponding gel with shorter running times, allowing for better visibility of the free-DNA bands. FOXP2 FHD concentrations are identically increased every four lanes, starting with no FHD, then repeated. Background YY1 is added in increasing concentrations in each box, with none present in the first box. The concentration of CNTNAP2 DNA is kept consistent throughout. FOXP2 FHD binds CNTNAP2 DNA in a clear distinct band present in the middle of the gel. YY1 interacts non-specifically with the DNA, particularly at higher YY1 concentrations, seen as a faint band at the top of the gel. This band disappears as the FOXP2 FHD concentration is increased because FOXP2 FHD likely outcompetes YY1 for the DNA. (**B**) Control gel to determine that YY1 does not specifically bind to CNTNP2 DNA.

In the second EMSA, (Figure 9), the effect of the presence of YY1 on the interaction between FOXP2 FHD and the FOXP2 cognate binding sequence (CNTNAP2 DNA) was investigated. FOXP2 FHD clearly forms a single FHD–DNA complex in the absence of YY1. Increasing concentrations of YY1 resulted in a new band at the top of the gel (Figure 9). This band was most intense in the absence of FOXP2 FHD and decreased in intensity as the FHD:DNA ratio increased, indicating competition taking place for the DNA by both proteins. As such, it can be concluded that the FOXP2 FHD does not bind to YY1 and DNA simultaneously. We can also conclude that the large YY1-CNTNAP2 DNA complex is due to the large YY1 oligomers binding non-specifically to the DNA, since no YY1-CNTNAP2 complexes could be observed on the smaller control gel (Figure 9B). This non-specific DNA binding of large YY1 species has been previously observed as a characteristic of YY1 [45]. This is unlike the variety of smaller complexes that YY1 forms with its specific DNA (Figure 8A).

## Discussion

### YY1 DNA binding and self-association

YY1 is an intrinsically disordered protein involved in a wide range of processes [22], including DNA structuring [33], transcriptional activation and repression [19,21,25], DNA repair [37] and mRNA processing [20]. Several (sometimes conflicting) mechanisms have been proposed in the literature to try to explain the dynamic activities of YY1 [19,20,30,33,45,47]. Among these mechanisms, and of most interest to this study, is the proposal that YY1 oligomerisation influences both DNA structuring and the specificity of DNA binding [33]. In this work, we have investigated in detail the relationship between YY1 oligomerisation and DNA binding by determining the effect of the electrostatic environment; the concentration of protein and DNA, and the effect of other protein–protein interactions on YY1 oligomerisation and dynamics. Additionally, we have attempted to relate our observations to YY1's properties as an intrinsically disordered protein with a known ability to undergo phase separation [19,21].

We observed an interesting dynamic between YY1 self-association and YY1–DNA interactions. The size of YY1 oligomers capable of interacting with DNA is dependent on the relative amount of DNA present. At low relative DNA concentrations, when YY1 is saturating, the average complex size increases, indicating that YY1 is capable of forming large oligomers on the DNA when YY1 is in excess (Figure 3). This is confirmed by both the anisotropy and EMSA (Figure 8) results. These results suggest that at least some of the YY1 multimeric interface consists of relatively weak, non-specific interactions typical of an intrinsically disordered protein [6,7]. On the other hand, when the DNA concentration is saturating, we observe a decrease in the average YY1 complex size, indicating a disruption of the oligomeric interface when DNA is in excess. This result also implies that the DNA binding domain of YY1 has some involvement in the YY1 self-association interface.

Unfortunately, the HDXMS peptide coverage of the YY1 DNA binding domain is too low to confirm any direct effect of YY1 oligomerisation on DNA binding. However, we have identified other regions of the sequence, upstream of the DNA binding domain, that change in dynamics upon DNA binding. In particular, the N-terminal acidic regions were shown to experience an increase in exposure to solution and likely an increase in disorder upon DNA binding (Figure 4). When taken in combination with the anisotropy and EMSA results, this increase in disorder is likely to be due to the dissociation of the oligomeric interface upon DNA binding. Indeed, previous speculation has suggested that the YY1 acidic regions may bind to the DNA-binding zinc finger domain, given their complementary charges and the involvement of the acidic region in YY1’s transcriptional activation capabilities [47]. Our observations confirm that this is likely to be the case. Furthermore, our results show evidence that there may be an oligomeric interface between the zinc fingers of YY1 and the acidic rich region.

**Figure 10:**
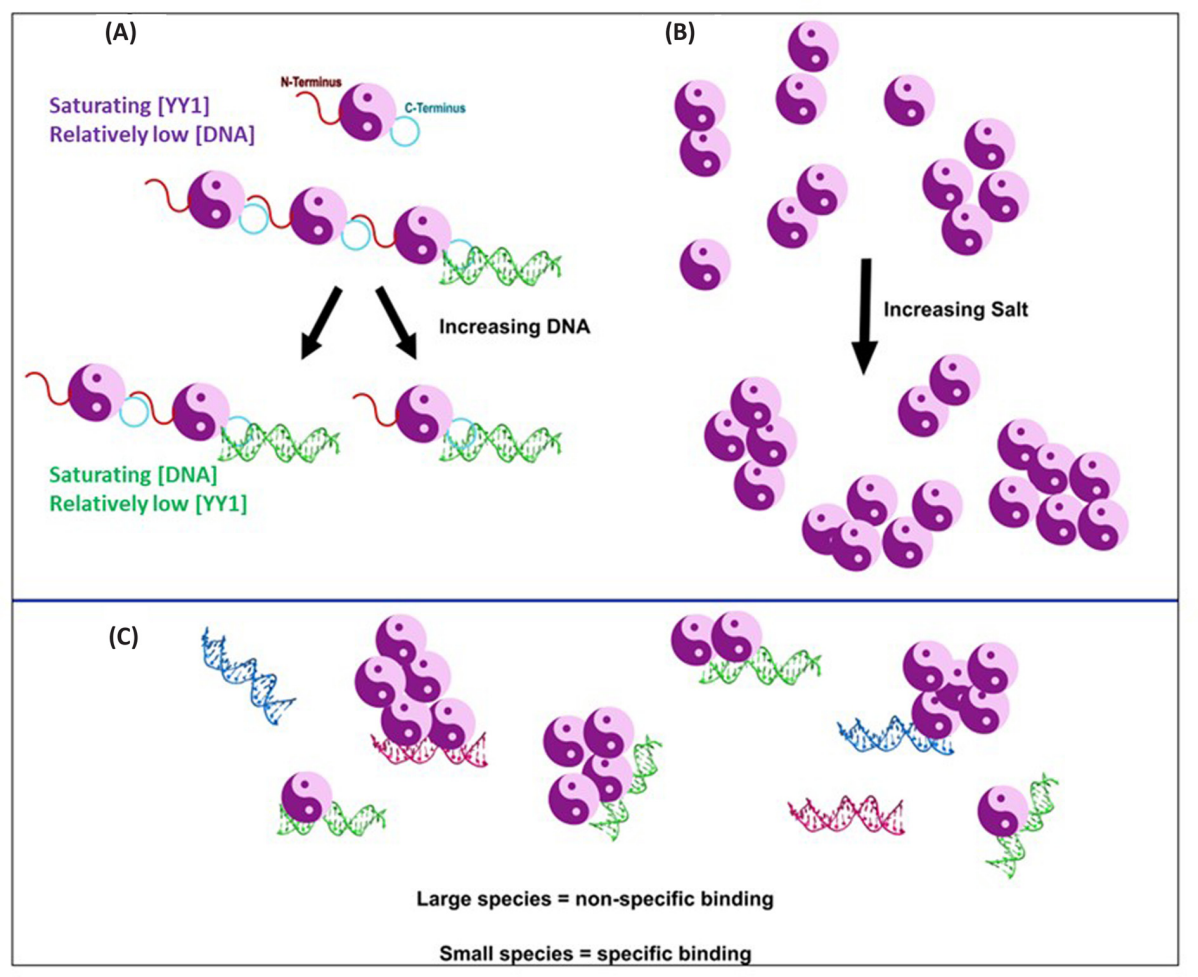
Model of how YY1 oligomerisation may be affected by DNA and ionic strength (**A**) The acidic N-terminal region binds to the zinc finger DNA binding domain on the C-terminal end of the protein to form oligomers. DNA also binds to the C-terminal end and can outcompete the acidic region for it. (**B**) With increased ionic strength, the average size of YY1 oligomers in solution increases. (**C**) Large oligomeric YY1 binds DNA with lowered specificity compared with smaller species. Image created using InkScape 1.0

From the YY1 oligomerisation behaviour observed, we propose a mechanism by which YY1 may form multimers in a DNA-dependent manner (Figure 10). YY1 molecules likely interact with each other via both the acidic region and the DNA-binding domain. This interaction is competed for by DNA, which binds to the DNA binding domain more strongly, breaking apart YY1 oligomers. However, when YY1 is at far higher concentrations compared to the DNA, the free acidic region of a YY1–DNA complex may interact with another free YY1 molecule’s DNA-binding domain, resulting in the formation of potentially large, multimeric YY1 species associated with a single DNA molecule (Figure 10). YY1 multimerisation may thus form an important aspect that regulates YY1 transcription through its association with DNA. Indeed, DNA containing the correct consensus sequence has been demonstrated to promote YY1 phase separation in the cell [19]. This process could also be dependent on competition with other proteins as well as on post translational modifications, which is a common trend with such regions in an intrinsically disordered protein [6].

The effect of ionic strength on the YY1 self-association interaction that is seen in this study supports the proposed manner by which the acidic region and DNA binding domain of YY1 interact, since ionic strength is more likely to influence charged interactions than those of non-polar origin [56]. Additionally, the observed shift in proportion of oligomeric species according to ionic strength (Figure 1) may allude to one of the mechanisms by which YY1 function is controlled. Very large YY1 multimers with weak intermolecular interactions are likely to be integral to the role YY1 plays in forming proteinaceous membrane-less organelles such as nuclear speckles and the polycomb complex. For these structures to form, high concentrations of protein are required to undergo a liquid–liquid phase transition, which is tightly controlled by factors such as post-translational modifications and salt concentrations [6]. The sensitivity of the YY1 oligomeric composition to salt concentration will contribute to this function. Additionally, the sensitivity of these processes to the exact conditions present may help to explain why in some studies, YY1 clearly forms different oligomers [33,37,45], while in others it only appears as a single species [46].

### YY1 DNA binding specificity

Previous studies have indicated that isolated YY1 oligomers seem capable of binding to DNA with altered specificity [37]. The binding behaviour of YY1 to FOXP2-specific CNTNAP2 DNA that we observed (Figure 9) confirms that YY1 is capable of binding to DNA with altered specificity depending on its oligomeric form. Our data indicate that YY1 is capable of binding non-specifically to CNTNAP2 DNA predominantly as a large multimeric complex. This is as opposed to the smaller complexes with which it also binds to YY1-specific DNA (Figures 8 and 9). This altered specificity for DNA depending on the oligomeric state of the protein, suggests an intriguing mechanism of control of transcription, where the affinity that YY1 has for any single DNA sequence is likely to be dependent on its multimeric state. In turn, the ability of YY1 to form these various multimers is tightly controlled by the concentration of YY1, the ionic strength and possibly also other factors such as post translational modifications. This means that it is possible for large changes in downstream processes to occur via only changing a single aspect of YY1’s macromolecular interactions. The ability of YY1 to bind non-specifically to different DNA sequences depending on its multimeric state could be linked to YY1’s role in proteinaceous membrane-less organelle formation, where in this way, the protein could bind to a large range of nucleic acids non-specifically, allowing the localisation of the necessary macromolecular components to such structures.

### YY1–FOXP2 interaction

YY1 and FOXP2 influence similar pathways, including neural development [27,61], and are co-localised in the cell [55]. One specific example is that both proteins are associated with transcriptional control of the CNTNAP2 gene, via interactions with the promoter region [50,62]. We ascertain here that YY1 and the FOXP2 FHD do interact with low affinity in isolation, as well as in the presence of DNA. Because this interaction is not influenced either by the presence of YY1-specific DNA or by ionic strength (Figure 7), it appears that the YY1-FOXP2 FHD interaction does not take place at the YY1 DNA binding domain, nor is it likely to be formed at the YY1 oligomeric interface at the N-terminal acidic region.

YY1 was found to be capable of binding to both the FOXP2 FHD and to its own YY1-specific DNA sequence at the same time. In contrast, the FOXP2 FHD is not able to bind simultaneously to its cognate DNA and YY1 and favours DNA over YY1 (Figure 9). Considering that YY1 can bind non-specifically to the FOXP2-specific CNTNAP2 binding sequence (Figure 9), and further considering that both YY1 and FOXP2 are associated with transcription of the CNTNAP2 gene [50,62], it is possible that both YY1 and FOXP2 are localised to similar regions within this promoter. This suggests that YY1 may play a part in FOXP2's DNA binding activity, or that both proteins may be involved in the formation of a much larger complex of transcription factors. Indeed, the fact that the YY1–FOXP2–DNA ternary complex only forms on YY1-specific DNA and not on FOXP2-specific DNA implies the significance of these interactions in specific regulatory processes. The interaction between these two proteins may well be relevant to the development of conditions such as autism spectrum and speech disorders and warrants further investigation.

## Conclusion

Our findings indicate that YY1 exists as a dynamic intrinsically disordered protein with what are likely to be multiple protein–protein binding sites occurring throughout the sequence (especially abundant in the acid-rich regions), which may also form small pockets of structure as a result. Some of these structured pockets become unbound and potentially disordered upon DNA binding, which suggests that these regions are molecular recognition features capable of gaining or losing structure upon binding events. This is supported for several of these regions by matching predicted MoRFs from the structure. Our results imply that YY1 likely self-associates at the acid-rich regions and possibly also at the DNA-binding domain. This finding links YY1 oligomerisation both to ionic strength and to DNA binding. We show that DNA is capable of binding to various oligomeric sizes of YY1 and that the size of the oligomeric species influences the specificity of DNA binding. The size of the oligomeric species is affected both by salt concentration and by DNA concentration. Increases in salt concentration increase the oligomeric size of YY1, which results in non-specific binding to DNA. On the other hand, low ionic strength and high DNA concentrations result in smaller sized oligomeric species that are capable of binding specifically to YY1-specific DNA. These findings provide much needed insight into YY1’s recently explored function in phase separation [19]. YY1 and FOXP2 FHD form a specific, fairly weak interaction both in isolation and on the YY1-specific DNA. This interaction does not happen at the YY1 DNA binding domain, nor does it occur at the oligomer interface at the acid rich regions. This interaction likely does happen at the DNA-binding region of the FOXP2 FHD, however, and therefore could be involved in regulating FOXP2 DNA-binding interactions. The interactions that YY1 forms with itself, DNA, and FOXP2, therefore, are part of a complex regulatory function which is essential for proper neural development. By better understanding the mechanisms discussed, it can be better understood how each transcription factor plays a role in regulating pathways related to conditions such as autism spectrum disorder, Schizophrenia and dementia.

## Methods and materials

### Protein expression and purification

Both the FOXP2 FHD and full-length YY1 constructs used in this study contained a hexahistidine tag at the N- terminal end. Each construct was inserted into a pET-11A plasmid (Genscript, U.S.A.), and used to transform T7 cells (Genscript, U.S.A.) as per the recommended protocol. FOXP2 FHD was expressed as previously described [63] at 20°C. YY1 was expressed under similar conditions, except that expression was induced with 0.2 mM IPTG and included 0.1 mM ZnCl_2_.

FOXP2 FHD and YY1 were purified using IMAC chromatography at pH 7.5 on a HiTrap column bound to nickel or cobalt ions respectively (Cytiva, U.S.A.), as described previously for FOXP2 FHD [63]. The FOXP2 FHD His-tag was removed with thrombin and separated using a benzamidine and HiTrap column in tandem [64]. The IMAC for YY1 used an alternative protocol, where the equilibration buffer had 500 mM NaCl, 50 mM imidazole and 20 mM Tris-HCl, followed by a salt and detergent wash and then an imidazole wash (500 mM NaCl, 100 mM imidazole, 20 mM Tris-HCl). YY1 was eluted by increasing the imidazole concentration to 800 mM. YY1 was further purified using size exclusion chromatography on a HiLoad Sephadex 16/600 200pg column (GE Heathlcare, U.S.A.). The solubility of all protein species was confirmed using absorbance at 340 nm. All protein aggregates/precipitates were removed from the sample prior to experiments.

### DNA sequences

Two double stranded DNA sequences were used in this study.

The first is specific to the FOXP2 FHD, and contains the FOXP2 core binding sequence with the flanking regions taken from the CNTNAP2 gene’s promoter region, since CNTNAP2 is a gene known to be regulated by FOXP2 [50]. This sequence is referred to in this study as CNTNAP2 DNA: 5′-TTTGATTGTTTACTTTGTTC-3′.

The second DNA sequence is YY1 specific. It originates from the Adeno-associated virus (AAV) P5 promoter, and has previously been used in the crystal structure of the YY1 DNA-binding domain [23]. YY1 binds to this specific sequence as part of its role in repressing expression of AAV genes [23]. This DNA sequence is referred to in this study as the AAV P5 promoter sequence: 5′-AGGGTCTCCATTTTGAAGCG-3′.

### Protein and DNA labelling

In order to perform the fluorescence anisotropy studies, it was necessary to label protein and/or DNA with a fluorescent dye. YY1 was labelled via the N-terminal hexahistadine tag with NTA ATTO 550 (ATTO Tech). This was achieved by incubating YY1 at 4°C overnight with a three times excess of NTA ATTO 550. Excess dye was removed using a Pierce™ dye removal column (ThermoFisher Scientific, U.S.A.) and the labelling efficiency was calculated by correcting the protein concentration for the label’s absorption with the following equation:

$$A_{protein}=A_{280}-(A_{554}/10)$$

DNA oligonucleotides used in fluorescence assays were purchased from IDT (Whitehead Scientific) already labelled with Rhodamine X (ROX) on the 5′ end of one strand.

### Anisotropy

Anisotropy assays were conducted so as to observe binding interactions between YY1 and its binding partners (YY1, FOXP2 FHD and DNA). They were performed by titrating increasing concentrations of the binding partner into fluorescently labelled YY1, or fluorescently labelled DNA. All anisotropy assays were performed in triplicate. The macromolecular interactions assessed were as follows:

DNA binding to YY1 labelled with NTA ATTO 550; YY1 binding to DNA labelled with ROX; YY1 binding to YY1 labelled with NTA ATTO 550; FOXP2 FHD binding to YY1 labelled with NTA ATTO 550; and FOXP2 FHD binding to YY1 labelled with NTA ATTO 550 when in the presence of 10 μM background YY1-specific DNA.

YY1 labelled with NTA ATTO 550 was used at a concentration of 0.2 μM, while labelled ROX DNA was used at 1 μM. The concentration of the unlabelled titrant was increased either until a stable asymptote was reached or until a point where higher stock concentrations resulted in protein aggregation. The added volume of the titrant did not exceed 10% of the initial sample volume, and each measurement was taken after allowing time for a binding equilibrium to be established. All assays were conducted in 100 mM NaCl and 10 mM HEPES, pH 7.5. YY1–YY1 and FOXP2 FHD-YY1 binding assays were also conducted in increased NaCl concentrations of 300 and 500 mM NaCl. All anisotropy binding assays were performed using a Perkin Elmer LS-50B Luminescence spectrometer connected to a LKB Bromma (2219 Mulitemp II) water bath set at 20°C, with excitation and emission slit widths set to 5 nm, and an integration time of 3 s. The excitation and emission wavelengths for ROX-DNA were 580 and 607 nm respectively, while for labelled YY1 they were 554 and 576 nm respectively. The G factor was calculated for each sample before addition of the titrant. Binding curves were analysed using the software SigmaPlot (version 12.0), where they were fit to the single site ligand binding model:

$$y=\frac{B_{max}x}{\left(K_{D}+x\right)}$$

Where *x* is the concentration of the ligand, *y* is the specific binding as observed with the relative anisotropy, *B*_max_ is the maximum number of binding sites and *K*_D_ is the concentration of ligand needed to reach half maximal binding. In the instances where multiple species were present in the binding isotherm, the true *K*_D_ could not be calculated. Although the exact mode of the ligand binding was unknown, this model proved the simplest interpretation of the data, and had the most reliable fit in comparison to alternative ligand binding models. Error bars in each curve represent the standard deviation between triplicate measurements.

To assess whether changes in NaCl concentration significantly affected the FOXP2 FHD-YY1 and the YY1–YY1 interactions, statistical analysis was done to compare the different curves. In both cases, the homogeneity of variance for the relative anisotropy values in each curve was tested using Bartlett and Levene’s tests. Normality for the relative anisotropy values was assessed with a Shapiro–Wilk test. As the FOXP2 FHD-YY1 interaction displayed both normality and equal variances (Bartlett, *P*=0.4214; Levene, *P*=0.3796; Shapiro–Wilk, *P*=0.05107), a one-way ANOVA was used to determine if there was a difference between the curves. In contrast, as the YY1–YY1 curves did not violate the criteria for a parametric test (Bartlett, *P* = 2.2e-16; Levene, *P* = 5.115e-8; Shapiro–Wilk, *P* = 1.09e-5), a Kruskal–Wallis test was used instead, followed by a post-hoc Dunn test. All statistical analyses were done using Rstudio. All *P*-values reported refer to these comparisons between conditions.

### Electrophoretic mobility shift assays (EMSA)

EMSAs were conducted to assess protein–DNA interactions qualitatively by providing information on the ability of the various YY1 oligomeric species to bind to DNA. In addition to confirming the DNA-binding activity, EMSA also provided information on the effect of the protein–protein interaction on YY1’s DNA-binding capacity. A constant concentration of free DNA was mixed with increasing concentrations of protein (originally in 100 mM NaCl) per sample, ranging from 0 to 5 μM of the protein. Additional lanes included the further addition of a second protein (either FOXP2 FHD or YY1) in order to probe the YY1–FHD–DNA interactions for each protein's specific DNA sequence. FOXP2 FHD was added to YY1-AAV P5 promoter DNA complexes at concentrations of 2.5, 5 and 10 μM. YY1 was added to FOXP2 FHD-CNTNAP2 DNA complexes at concentrations of 2.5, 5 and 7.5 μM. Samples consisted of 0.5 μM DNA, protein and 20% (v/v) binding buffer (10 mM HEPES, pH 7.5, 1 mM MgCl_2_, 100 mM KCl, 10% (w/v) glycerol and 0.1 mg/ml BSA). Samples were left overnight at 4°C to allow for full binding equilibrium to be reached. A 10% polyacrylamide gel was prepared, as previously described [65] using a Bio-Rad (USA) PROTEAN™ II xi Cell gel cassette. The gels were run in 1 x TBE buffer, at 4°C and 160 V for 6 or 8 h. A 10 000 times dilution of GelRed® (Biotium) was used to view the resulting bands. Further control gels were used to ensure non-specificity for binding of YY1 to CNTNAP2 DNA and FOXP2 FHD to AAV P5 DNA. These were prepared as detailed above, but resolved with a Bio-Rad (USA) mini-PROTEAN Tetra Cell.

### Hydrogen-deuterium exchange mass spectrometry (HDX-MS)

The dynamics of YY1’s structure in both the apo and the DNA bound (1:1 DNA:protein in mg/ml, coming to 3× molar excess) forms was probed with HDX-MS using an automated LEAP PAL HDX system (Leap Technologies) coupled to an Agilent 1100 HPLC system and Sciex 6600 TripleTOF mass spectrometer. Three labelling time points: 15, 600 and 3600 s were used. Five micrograms of protein in a 10 mM HEPES buffer, pH 7.5, with 100 mM NaCl was diluted in D_2_O to a final concentration of 90% at 20°C to initiate labelling. The exchange reaction was then quenched with a two-fold dilution of 3 M guanidinium chloride and 20 mM TCEP, pH 2.5 at 0°C. Non-deuterated and fully deuterated controls were utilised. For full deuteration, proteins were incubated overnight (18 hrs) in D_2_O at a 90% final concentration. Quenched samples were digested online by passing each through a 30 × 2.1 mm Poroszyme pepsin column (Applied Biosystems, U.S.A.) at 100 μl/min for an incubation time of ∼60 s, followed by desalting on a Acclaim PepMap (Thermo Scientific, U.S.A.) trap column (LC packing, ID:1.0 mm, phase C18) and then separated using an analytical column of 50 × 2.1 mm C18 (Aeris Peptide 3.6 μm particle size) directly coupled to a TurboIon ESI source of an AB Sciex 6600 TripleTOF mass spectrometer (AB Sciex, U.S.A.). Peptides were eluted using an acetonitrile gradient of 10–25% for 10 min at a flow rate of 200 μl/min All columns, pepsin, trap and analytical were kept at 4°C in a temperature controlled column oven. All materials used for the hydrogen exchange experiment are indicated in Supplementary Table S1.

For peptide identification the 6600 TripleTOF was operated in Data Dependant Acquisition (DDA) mode while for deuterium labelling only a precursor scan was collected. In DDA mode precursor scans were acquired from *m*/*z* 360 to 1500 using an accumulation time of 250 ms followed by 30 product scans, acquired from *m*/*z* 100 to 1800 at 100 ms each, for a total scan time of 3.3 s. Charge ions (1- 5, that fall in the mass range 360e1500 *m*/*z*) were automatically fragmented in Q2 collision cells using nitrogen as the collision gas. Collision energies were chosen automatically as function of m/z and charge. Dynamic exclusion was set to 15 s. HDX-MS experiments were done in triplicate

PEAKS Studio version 6 was used to identify peptide peaks and their associated properties, including their sequences, retention times, charge information and peptide quality scores. The peptide pool produced was imported into the analysis program HD Examiner (Sierra Analytics, U.S.A.), which then calculated deuterium uptake for each peptide per time point measured. Most peptides displayed characteristic EX2 uptake behaviour. Those which displayed EX1 were set to low confidence. Further information is provided in Table 1. The mass spectrometry proteomics data have been deposited to the ProteomeXchange Consortium via the PRIDE [66] partner repository with the dataset identifier PXD038431.

**Table 1:** HDXMS experimental conditions and peptide information

Dataset	Apo	DNA-bound
**Labelling conditions:**	100 mM N1aCl; 2 mM DTT; 10 mM HEPES; pH 7.5; 20°C	100 mM NaCl; 2 mM DTT; 10 mM HEPES; pH 7.5; 20°C
**Percent D2O**	90	90
**Time course (s)**	15; 600; 3600	15; 600; 3600
**Controls**	Fully deuterated sample (Apo)	Fully deuterated sample (DNA-bound)
**Back exchange % (mean; IQR)**	44.43; 21.3	43.65; 19.1
**Number of peptides**	124	107
**Sequence coverage (% amides covered by peptides)**	83.2%	83.2%
**Average peptide length/Redundancy**	14.6/0.35	14.3/0.31
**Peptide redundancy (total peptide/total amides)**		
**Replicates (biological or technical)**	3 biological	3 biological
**Repeatability (e.g. average stdv of deuterium percent)**	7.18	3.39
**Significant differences in HDX (change in deuterium uptake)**	≥ 30%	≥ 30%

### Size exclusion chromatography (SEC)

To better understand the oligomeric states of YY1, size exclusion high performance liquid chromatography (SE-HPLC) was performed at two NaCl concentrations: 300 and 500 mM NaCl. YY1 species were separated using a 4.6 mm × 300 mm Yarra 3μm SEC-3000 SE-HPLC (Phenomenex, U.S.A.) column connected to a SHIMADZU SP20A HPLC instrument (Japan). Samples of 20 μl protein were resolved in a buffer of 20 mM HEPES, pH 6.8 and either 300 or 500 mM NaCl (lower salt concentrations were not applicable as they resulted in non-specific interactions with the column). Absorbance values of the eluent were detected simultaneously at 254 and 280 nm and a flow rate of 0.35 ml/min was used.

## Data Availability

The authors agree to make any relevant materials, data, code and associated protocols available on request. HDX data is available in the PRIDE partner repository with the dataset identifier PXD038431. Account details for reviews are as follows: Username: reviewer_pxd038431@ebi.ac.uk; Password: dynpFQj2.

## Abbreviations

ASD: autism spectrum disorder
DBD: DNA binding domain
FHD: forkhead domain
FOXP2: Forkhead box P2
HDX-MS: hydrogen-deuterium exchange mass spectrometry
IDP: intrinsically disordered protein
PMO: proteinaceous membrane-less organelles
SE-HPLC: size exclusion high performance liquid chromatography
SEC: size exclusion chromatography
YY1: Yin Yang 1
